# Profil épidémiologique des épicondylites latérales en milieu de rééducation

**DOI:** 10.11604/pamj.2020.36.265.21403

**Published:** 2020-08-11

**Authors:** Mouna Sghir, Takieddine Elhersi, Anouer Abdallah, Aymen Haj Salah, Nadia El Khemiri, Nabil Dammak, Wassia Kessomtini

**Affiliations:** 1 Service de Médecine Physique et de Réadaptation Fonctionnelle, EPS Tahar Sfar Mahdia, Mahdia, Tunisie,; 2 Hôpital Régional de Tozeur, Tozeur, Tunisie,; 3 Service d’Orthopédie, EPS Tahar Sfar Mahdia, Mahdia, Tunisie

**Keywords:** Epicondylite latérale, épidémiologie, rééducation fonctionnelle, Lateral epicondylitis, epidemiology, functional rehabilitation

## Abstract

**Introduction:**

l’épicondylite latérale, mieux connue sous le nom de « Tennis elbow », fait partie des troubles musculo-squelettiques du membre supérieur et constitue un problème majeur de santé publique. Le but de notre étude est d'évaluer le profil épidémio-clinique et les modalités thérapeutiques des patients suivis au service de médecine physique et réadaptation, pour épicondylite latérale.

**Méthodes:**

il s'agit d'une étude rétrospective sur 6 ans (2012-2017) portant sur des patients adressés au Service de Médecine Physique et de Réadaptation Fonctionnelle au CHU Tahar Sfar Mahdia pour une épicondylite latérale. Les caractéristiques sociodémographiques et cliniques ainsi que les modalités thérapeutiques ont été évalués pour chaque patient.

**Résultats:**

cinquante patients ont été inclus. L’âge moyen était de 44,82 ans, avec une prédominance féminine (72%). La moitié de nos patients étaient actifs et la majorité (60%) effectuait un travail type bureautique. Le membre supérieur droit était le plus touché dans 78% des cas. La durée moyenne des symptômes était de 14,66 mois. L’examen clinique a révélé une triade tendineuse positive, au niveau des épicondyliens latéraux dans 96% des cas. L’examen radiologique a été réalisé pour 8 patients, et l’échographie pour 6 patients. Tous les patients ont reçu un traitement antalgique, 84% des patients ont reçu des anti-inflammatoires non stéroïdiens et seulement 8 patients ont bénéficié d’une infiltration de corticoïdes. Un seul patient a bénéficié d’un traitement chirurgical après échec de la prise en charge médicale. Des séances de rééducation ont été prescrites chez 92% des patients. Une amélioration totale a été notée chez 42% des patients, 46% ont rapporté une amélioration transitoire et 12% ont évolué vers la chronicité.

**Conclusion:**

l’épicondylite latérale est une source fréquente de douleur du coude. Sa prise en charge en Médecine Physique repose sur un traitement médical et une rééducation fonctionnelle adaptée. Mais aucune option thérapeutique ne semble être clairement supérieure à l’autre.

## Abstract (en)

**Introduction:**

lateral epicondylitis, better known as tennis elbow, falls within the spectrum of upper extremity musculoskeletal disorders and it is a major public health problem. The purpose of our study was to determine the epidemiological and clinical profile and treatment modalities for patients with lateral epicondylitis followed at the Department of Physical Medicine and Rehabilitation.

**Methods:**

we conducted a retrospective study (2012-2017) of patients with lateral epicondylitis referred to the Department of Physical Medicine and Functional Rehabilitation at the Tahar Sfar Mahdia University Hospital over a period of 6 years. Socio-demographic and clinical features as well as treatment modalities were assessed for each patient.

**Results:**

fifty patients were enrolled in the study. The average age of patients was 44.82 years, with predominance of the female sex (72%). Half of our patients were active and the majority of them (60%) worked in a traditional office environment. Right upper extremity was most affected (78% of cases). The median duration of symptoms was 14.66 months. Clinical examination showed positive tendon triad in the lateral epicondyle in 96% of cases. Radiological examination was performed in 8 patients, ultrasound in 6 patients. All patients received analgesics, 84% of patients received non-steroidal anti-inflammatory drugs, only 8 patients received corticosteroid injections. One patient underwent surgery after therapeutic failure. Ninety-two percent of patients were scheduled for rehabilitation sessions. Total improvement was noted in 42% of patients, 46% reported transient improvement and 12% progressed to chronicity.

**Conclusion:**

lateral epicondylitis is a common cause of elbow pain. Its management in physical medicine is based on medical treatment and appropriate functional rehabilitation. However no therapeutic option seems clearly superior to the other.

## Introduction

L’épicondylite latérale, mieux connue sous le nom de «Tennis elbow», fait partie des troubles musculo-squelettiques (TMS) du membre supérieur. Cette pathologie qui résulte de l’hyper sollicitation des muscles extenseurs, aboutissant à l’inflammation ou l’irritation des insertions tendineuses [1] se voit dans la plus grande portion chez les travailleurs. Sa survenue augmente avec l’âge, prédominant entre 40 et 60 ans avec un pic vers 45 et 54 ans [2]. Ainsi, elle constitue un problème majeur de santé publique, vu qu’elle entraine une dégradation de la qualité de vie, une perte d’emploi et un coût économique élevé [3]. Le diagnostic de l’épicondylite latérale est exclusivement clinique, fondé sur les données de l’interrogatoire et de l’examen clinique. Le traitement de première intention est basé sur le repos et le traitement médico-rééducatif. Malgré la fréquence de cette affection qui ne cesse de croitre et ses lourdes conséquences, peu d’études ont été menées sur ce sujet notamment en milieu de rééducation. Notre travail avait donc pour objectifs: d’étudier les caractéristiques épidémio-cliniques des patients suivis pour épicondylites latérales en milieu de rééducation et de déterminer les modalités thérapeutiques ainsi que les aspects évolutifs.

## Méthodes

Il s’agit d’une étude rétrospective et descriptive. Elle a concerné les patients suivis au service de Médecine Physique et Réadaptation Fonctionnelle du CHU Taher Sfar Mahdia (Tunisie) pour épicondylite latérale, et ceci sur une période de 6 ans (de janvier 2012 à décembre 2017).

**Critères d’éligibilité et recueil des données:** ont été inclus dans notre étude les patients de deux sexes et pour lesquels le diagnostic d’une épicondylite latérale a été retenu. Les caractéristiques épidémiologiques, cliniques, paracliniques, les modalités thérapeutiques et évolutives pour chaque patient ont été recueillies.

**Analyse statistique:** les données ont été saisies à l’aide d’un logiciel SPSS pour Windows (version 21). Les résultats ont été exprimés par la moyenne ± l’écart type pour les variables quantitatives et par les pourcentages pour les variables qualitatives.

## Résultats

Nous avons colligé 50 patients: 36 femmes (72%) et 14 hommes (28%), d’âge moyen de 44,82 ± 7,07 ans avec des extrêmes de 27 à 59 ans. La tranche d’âge [35-59 ans] était la plus fréquemment touchée dans 46% des cas. La majorité de nos patients (54%) n’avait aucun antécédent pathologique notable. Le diabète a été noté dans 4% des cas et l’hypertension artérielle (HTA) dans 2% des cas. Le délai moyen du diagnostic de l’épicondylite était de 14,66 ±20,59 mois avec des extrêmes de 1 à 84 mois. La moitié de nos patients étaient actifs et la majorité (60%) effectuait un travail de type bureautique. Le travail forcé a été objectivé chez 10 patients (20%) et l’utilisation d’outils vibrants chez 6 patients (12%) (Tableau 1). L’ancienneté professionnelle moyenne des travailleurs au poste de travail a été de 13,65±10,39 ans avec des extrêmes de 1 à 30 ans. Les facteurs favorisants mis en évidence, dans notre étude, sont résumés dans le Tableau 2. Concernant le devenir professionnel, vingt-deux patients (44%) ont bénéficié d’un aménagement du poste de travail, 8 patients (16%) ont perdu leur emploi et 20 patients (40%) ont été mis à la retraite anticipée.

**Tableau 1:** caractéristiques professionnelles de la population d’étude

Poste de travail	Nombre	Pourcentage (%)
Bureautique	30	60
Travail forcé	10	20

**Tableau 2:** les facteurs favorisants de l’épicondylite latérale

Facteurs favorisants	Nombre	Pourcentage (%)
Répétitivité des gestes	13	26
Travail forcé	10	20
Utilisation d’outils vibrants	6	12

Les signes fonctionnels ont été dominés par les douleurs du coude chez tous les patients, associées à une gêne fonctionnelle dans 80% et une impotence fonctionnelle dans 16% des cas (Tableau 3). L’épicondylite était du côté droit dans 39 cas (78%), gauche dans 10 cas (20%) et bilatérale dans 1 seul cas (2%). L’Intensité moyenne de la douleur mesurée par L'échelle visuelle analogique (EVA) était de 5,5cm. L’examen clinique a révélé des douleurs à la palpation de l’épicondyle latéral ainsi qu’à la contraction résistée chez tous les patients, et à l’étirement des épicondyliens dans 96% des cas. La radiographie standard du coude a été réalisée chez seulement 8 patients (16%): elle était normale dans 7 cas et elle mettait en évidence une calcification des parties molles chez 1 seul patient. L’échographie des parties molles a été pratiquée chez 6 patients (12%). Elle objectivait un épaississement tendineux hypo-échogène dans 2 cas (4%), une calcification intra-tendineuse dans 2 cas (4%), et elle était normale pour les deux derniers cas.

**Tableau 3:** la répartition des patients selon les signes fonctionnels

Signes fonctionnels	Nombre	Pourcentage (%)
Douleur du coude	50	100
Gêne fonctionnelle	40	80
Impotence fonctionnelle	8	16

Sur le plan thérapeutique, tous les patients ont reçu un traitement antalgique; le paracétamol étant l’antalgique le mieux toléré, il a été prescrit de première intention chez tous les patients et le recours à la prescription d’un traitement antalgique de palier II à dose réduite a été envisagé dans 2% des cas. Les anti-inflammatoires non stéroïdiens (AINS) ont été prescrits chez 84% des patients en cure courte et seulement 8 patients ont bénéficié d’une infiltration de corticoïdes. La rééducation fonctionnelle a été prescrite chez 92% de nos patients avec un nombre moyen de séances de 16,35. L’appareillage à type d’orthèse n’a été prescrit que pour deux patients (4%). Un seul patient a bénéficié d’un traitement chirurgical après échec du traitement médico-rééducatif et un passage à la chronicité. L’évolution chez nos patients a été marquée par une amélioration totale avec disparition durable de la douleur et récupération intégrale et permanente de la capacité fonctionnelle dans 42% des cas, une amélioration transitoire dans 46% des cas et une évolution vers la chronicité dans 12% des cas.

## Discussion

Dans cette étude nous avons noté que l’épicondylite était plus fréquente chez les sujets actifs de la quarantaine de sexe féminin et effectuant essentiellement un travail type bureautique. L’examen clinique révélant une triade tendineuse positive au niveau des épicondyliens latéraux a permis le diagnostic dans 96% des cas. Pour la prise en charge, elle était essentiellement médico-rééducative permettant une amélioration totale chez 42% des patients. Dans notre série, l’âge moyen était de 44,82 ± 7,07 ans avec des extrêmes allant de 27 à 59 ans; ceci est concordant avec l’étude de Kacem *et al*. [4], où l’âge moyen au moment de diagnostic était de 43,37 ± 7,3 ans avec des extrêmes allant de 24 à 61 ans. Dans la littérature, il existe une variabilité de l’incidence de la maladie selon l’âge. Sanders *et al*. [5], ont rapporté que l’incidence chez les travailleurs moins de 40 ans était plus importante que chez les plus âgés. Cependant, dans l’étude de Degen *et al*. [6], cette incidence était élevée chez les patients âgés de 65 ans ou plus. D’autres part, l’association entre le genre et l’épicondylite latérale est encore controversée [7-9]; la prédominance féminine de la maladie a été rapportée dans notre série, avec un sexe ratio de 0,39. Cette constatation a été confirmée par Kacem *et al*. [4] avec un sexe ratio de 0,21. Ceci peut s’expliquer par le fait que les hommes occupent souvent, des postes où l’activité physique mobilise tout le corps, alors que les femmes ont des postes où les gestes sont plus souvent répétitifs ou statiques. De plus, il arrive que les postes de travail ne soient pas adaptés à l’occupation par une femme, car conçus à l’origine pour des hommes [10]. Enfin, il semble que selon des études récentes, [11,12] les hormones influencent la perception de la douleur; par effet protecteur de la testostérone associé à un faible taux d’œstrogène et par la variation cyclique de la douleur pour les femmes en période d’activité génitale (activation du système nociceptif).

Concernant le diagnostic positif, le délai moyen dans notre étude était de 14,66 ± 20,59 mois avec des extrêmes de 1 à 84 mois. Ce délai est variable selon les séries [13], cela peut être expliqué par toute la composante bio-psycho-sociale qui entre dans le cadre de l’évaluation de la douleur, aussi bien par le patient que par le soignant. La moitié de nos patients étaient actifs et la majorité (60%) effectuait un travail de type bureautique. En France, la prévalence de l’épicondylite dans la population générale est de 0,7 à 4%. Cependant en milieu professionnel, elle est de 4 à 30% en fonction des divers secteurs d’activités [1]. D’autre part, plusieurs études ont mis en évidence des facteurs biomécaniques de risques majeurs de TMS-MS comme l’exposition aux gestes répétitifs [14], l’utilisation d’outils vibrants [15], le travail forcé, le travail au froid et l’éclairage inadéquat [16]. Dans notre série, on a constaté la présence de certains de ces facteurs de risque, telle qu’une répétitivité des gestes dans 26%, un travail forcé chez 10 patients (20%) et une utilisation d’outils vibrants chez 6 salariés (12%). Vu que l’adaptation ou le changement de poste de travail est souvent soit impossible, soit insuffisant pour mettre au repos le coude, l’arrêt du travail est souvent nécessaire. Cependant, la perte d’emploi à cause des épicondylites latérales professionnelles parait faible comparativement aux autres TMS [17]. En effet, l’arrêt de travail été noté dans 16% des cas dans notre série. Une étude réalisée en France a noté que seulement 9 salariés souffrant d’épicondylite latérale professionnelle ont perdu leur travail sur un total de 273 des salariés qui ont quitté leur travail pour une autre TMS [17]. Sur le plan clinique, l’examen physique toujours comparatif, cherche les trois signes cardinaux de la tendinite: la douleur spontanée par la pression, à l’étirement passif et enfin lors de la contraction active de l’élément concerné contre résistance [18]. Dans notre étude, on notait une douleur à la palpation de l’épicondyle ainsi qu’au test de contraction résistée chez tous les patients, et lors des manœuvres d’étirement dans 96% des cas. Lorsque tous les signes cliniques sont présents, un bilan complémentaire n’est pas indispensable. Cependant, devant un tableau clinique atypique ou pour éliminer d’autres pathologies, le thérapeute peut demander un bilan d’imagerie [19]. La radiographie standard est le plus souvent normale et met rarement en évidence la présence des calcifications des parties molles (intra-tendineuses), qui ont été objectivées dans un seul cas de notre série, et d’éliminer une pathologie articulaire [19]. L’échographie confirme le diagnostic en précisant le siège et la gravité de la lésion.

Le traitement d’une épicondylite latérale est avant tout médical. Il est toujours associé à un traitement fonctionnel et préventif pour éviter les rechutes [20]. L’objectif thérapeutique est de guider le processus de cicatrisation tendineuse. Le traitement chirurgical reste controversé, parce que le traitement conservateur permet une guérison dans plus de 90% des cas et qu’il existe un grand nombre de techniques chirurgicales dont aucune n’a démontré son efficacité par rapport aux autres [5]. L’évolution des épicondylites latérales peut s’avérer longue, entre 9 et 24 mois avec une moyenne de 12 mois. Pendant ces 12 mois elle peut entraîner des difficultés à réaliser le travail et les tâches de la vie quotidienne. En effet, une étude réalisée par Descatha *et al*. [14] sur les épicondylites latérales professionnelles, une évolution était favorable dans environ deux tiers des cas. Cette évolution dépend de la précocité du diagnostic et de la prise en compte des facteurs professionnels dans la prise en charge. Ainsi, la persistance de l’exposition aux travaux sollicitant le coude constitue un facteur de non-guérison. Ce fait explique le nombre non négligeable de patients ayant perdu leur travail dans la présente enquête puisque le changement de poste de travail n’était pas possible. En effet, le médecin de travail fait face souvent à des difficultés pour le reclassement professionnel des salariés qui ne sont pas qualifiés.

Plusieurs limites devraient être prises en considération dans l’interprétation des données issues de notre travail: le caractère rétrospectif de notre étude, le manque d’uniformité et d’exhaustivité des informations disponibles sur la base des données peuvent être à l’origine de certains biais de sélection; le faible effectif des patients (50 cas) ne permet pas de tirer des conclusions fiables sur la fréquence de la maladie, ni sur les différentes manifestations cliniques ou les caractéristiques évolutives; la sous-déclaration des plaintes, qui peut être liée au défaut de diagnostic, à la méconnaissance de la réglementation par les médecins traitants ou les médecins ayant posé le diagnostic, une crainte pour l'aptitude à l’emploi ou aux spécificités des systèmes officiels de déclaration des maladies professionnelles, ne donne qu’une idée approximative de l’incidence des épicondylites externes professionnelles; l’absence de précisions sur les raisons de la cessation du travail, nous laisse penser que d’autres facteurs puissent jouer un rôle.

## Conclusion

L’épicondylite latérale, dont la prévalence reste sous-estimée, est responsable d’impacts socio-professionnels importants. Cette constatation justifie l’importance du diagnostic et l’identification des facteurs de risque, afin de prendre rapidement les mesures préventives adéquates tant au niveau individuel que collectif et d’agir avant la survenue de cette affection tout en préservant le maintien de l’emploi. Concernant sa prise en charge en Médecine Physique, elle repose sur un traitement médical et une rééducation fonctionnelle adaptée. Mais aucune option thérapeutique ne semble être clairement supérieure à l’autre.

### Etat des connaissances sur le sujet

- L’épicondylite latérale fait partie des troubles musculo-squelettiques du membre supérieur très fréquents en milieu professionnel;
- L’épicondylite latérale constitue un problème majeur de santé publique.

### Contribution de notre étude à la connaissance

- Le profil épidémio-clinique et thérapeutique de l’épicondylite latérale en milieu de rééducation n’a pas été étudié avant.

## Remerciements

Je veux exprimer ma gratitude pour tous les co-auteurs et les reviewers.

## References

[1] Walz DM, Newman JS, Konin GP, Ross G (2010). Epicondylitis: pathogenesis, imaging, and treatment. Radiographics.

[2] Tosti R, Jennings J, Sewards JM (2013). Lateral epicondylitis of the elbow. AM J Med.

[3] Staal JB, de Bie RA, Hendriks EJ (2007). Aetiology and management of work-related upper extremity disorders. Best Pract Res Clin Rheumatol.

[4] Kacem I, Kalboussi H, Maoua M, El Guedri S, Brahem A, Boughattas W (2017). Les épicondylites latérales professionnelles dans la région du centre Tunisien: épidémiologie et devenir professionnel. Arch Mal Prof Enviro.

[5] Sanders TL, Maradit Kremers H, Bryan AJ, Ransom JE, Smith J, Morrey BF (2015). The epidemiology and health care burden of tennis elbow: a population-based study. Am J Sports Med.

[6] Degen RM, Conti MS, Camp CL, Altchek DW, Dines JS, Werner BC (2018). Epidemiology and Disease Burden of Lateral Epicondylitis in the USA: Analysis of 85,318 Patients. HSS J.

[7] Haahr JP, Andersen JH (2003). Physical and psychosocial risk factors for lateral epicondylitis: a population based case-referent study. Occup Environ Med.

[8] Fan ZJ, Silverstein BA, Bao S, Bonauto DK, Howard NL, Smith CK (2014). The association between combination of hand force and forearm posture and incidence of lateral epicondylitis in a working population. Hum Factors.

[9] Herquelot E, Gueguen A, Roquelaure Y, Bodin J, Serazin C, Ha C (2013). Work-related risk factors for incidence of lateral epicondylitis in a large working population. Scand J Work Environ Health.

[10] Fillingim RB, King CD, Ribeiro-Dasilva MC, Rahim-Williams B, Riley JL (2009). Sex, gender, and pain: a review of recent clinical and experimental findings. J Pain.

[11] Paller CJ, Campbell CM, Edwards RR, Dobs AS (2009). Sex-based differences in pain perception and treatment. Pain Med.

[12] Cairns BE, Gazerani P (2009). Sex-related differences in pain. Maturitas.

[13] Taanila H, Suni J, Pihlajamaki H, Mattila VM, Ohrankammen O, Vuorinen P (2009). Musculoskeletal disorders in physically active conscripts: a one-year follow-up study in the Finnish Defence Forces. BMC Musculoskelet Disord.

[14] Descatha A, Herquelot E, Mediouni Z, Petit A, Ha C, Leclerc A (2014). Épicondylalgies latérales dans une cohorte de salariés ligériens: évolution et déterminants. Rev Rhum Ed Fr.

[15] Hagberg M (2002). Clinical assessment of musculoskeletal disorders in workers exposed to hand-arm vibration. Int Arch Occup Environ Health.

[16] Piedrahíta H, Punnett L, Shahnavaz H (2004). Musculoskeletal symptoms in cold exposed and non-cold exposed workers. Int J Ind Ergon.

[17] Serazin C, Ha C, Bodin J, Imbernon E, Roquelaure Y (2013). Employment and occupational outcomes of workers with musculoskeletal pain in a French region. Occup Environ Med.

[18] Waseem M, Nuhmani S, Ram CS, Sachin Y (2012). Lateral epicondylitis: a review of the literature. J Back Musculoskelet Rehabil.

[19] Franceschi L, Boisaubert B, Rodineau J, Hérisson C (2006). Lesions des tendons et bourses séreuses. Le coude microtraumatique.

[20] Hong QN, Durand MJ, Loisel P (2004). Treatment of lateral epicondylitis: where is the evidence?. Joint Bone Spine.

